# Supplementary material for: Multi‐dataset identification of innovative feature genes and molecular mechanisms in keratoconus
Source: J Cell Mol Med. 2024 Sep 19;28(18):e70079. doi: 10.1111/jcmm.70079 (PMC11412914; doi:10.1111/jcmm.70079)
Supplement: Supplementary file 2 — Material S2. [file JCMM-28-e70079-s002.zip › 7.GSEA analysis/1.ARL11/KEGG_ABC_TRANSPORTERS.html]

Details for gene set KEGG\_ABC\_TRANSPORTERS[GSEA]

|  || Dataset | ARL11.group.cls#h\_versus\_l |
| Phenotype | group.cls#h\_versus\_l |
| Upregulated in class | h |
| GeneSet | KEGG\_ABC\_TRANSPORTERS |
| Enrichment Score (ES) | 0.3035949 |
| Normalized Enrichment Score (NES) | 0.9120546 |
| Nominal p-value | 0.5566219 |
| FDR q-value | 1.0 |
| FWER p-Value | 1.0 |
Table: GSEA Results Summary

  

Fig 1: Enrichment plot: KEGG\_ABC\_TRANSPORTERS      
 Profile of the Running ES Score & Positions of GeneSet Members on the Rank Ordered List

  

| SYMBOL | TITLE | RANK IN GENE LIST | RANK METRIC SCORE | RUNNING ES | CORE ENRICHMENT || 1 | ABCC9 | na | 627 | 0.451 | 0.0575 | Yes |
| 2 | ABCB1 | na | 668 | 0.442 | 0.1304 | Yes |
| 3 | ABCG5 | na | 1770 | 0.313 | 0.1511 | Yes |
| 4 | ABCB5 | na | 2070 | 0.292 | 0.1914 | Yes |
| 5 | ABCB11 | na | 2810 | 0.249 | 0.2119 | Yes |
| 6 | ABCD4 | na | 3044 | 0.239 | 0.2451 | Yes |
| 7 | ABCA3 | na | 3082 | 0.237 | 0.2838 | Yes |
| 8 | ABCA1 | na | 3637 | 0.214 | 0.3036 | Yes |
| 9 | ABCA7 | na | 5475 | 0.152 | 0.2762 | No |
| 10 | ABCA9 | na | 5622 | 0.148 | 0.2967 | No |
| 11 | ABCG1 | na | 6605 | 0.121 | 0.2888 | No |
| 12 | ABCA4 | na | 7986 | 0.093 | 0.2645 | No |
| 13 | ABCA5 | na | 8094 | 0.091 | 0.2766 | No |
| 14 | ABCC5 | na | 9269 | 0.071 | 0.2546 | No |
| 15 | ABCB8 | na | 9339 | 0.070 | 0.2643 | No |
| 16 | ABCC10 | na | 10112 | 0.059 | 0.2519 | No |
| 17 | ABCC2 | na | 10173 | 0.058 | 0.2599 | No |
| 18 | ABCA12 | na | 10215 | 0.058 | 0.2684 | No |
| 19 | ABCB7 | na | 12295 | 0.033 | 0.2140 | No |
| 20 | ABCB10 | na | 12874 | 0.028 | 0.2021 | No |
| 21 | ABCA10 | na | 13395 | 0.024 | 0.1911 | No |
| 22 | TAP2 | na | 16908 | -0.003 | 0.0903 | No |
| 23 | ABCA6 | na | 17170 | -0.004 | 0.0835 | No |
| 24 | ABCD3 | na | 17290 | -0.005 | 0.0809 | No |
| 25 | ABCB9 | na | 18229 | -0.012 | 0.0559 | No |
| 26 | ABCA2 | na | 18660 | -0.015 | 0.0459 | No |
| 27 | ABCB6 | na | 18935 | -0.017 | 0.0408 | No |
| 28 | TAP1 | na | 20591 | -0.028 | -0.0022 | No |
| 29 | ABCB4 | na | 21193 | -0.033 | -0.0140 | No |
| 30 | ABCC12 | na | 21670 | -0.037 | -0.0216 | No |
| 31 | ABCC11 | na | 22756 | -0.048 | -0.0450 | No |
| 32 | ABCG4 | na | 25549 | -0.084 | -0.1115 | No |
| 33 | ABCD1 | na | 25594 | -0.084 | -0.0986 | No |
| 34 | ABCC3 | na | 26009 | -0.090 | -0.0954 | No |
| 35 | ABCA8 | na | 26673 | -0.102 | -0.0974 | No |
| 36 | ABCC1 | na | 27080 | -0.110 | -0.0907 | No |
| 37 | CFTR | na | 27887 | -0.128 | -0.0925 | No |
| 38 | ABCC6 | na | 27956 | -0.130 | -0.0727 | No |
| 39 | ABCD2 | na | 29308 | -0.166 | -0.0839 | No |
| 40 | ABCC8 | na | 29784 | -0.181 | -0.0673 | No |
| 41 | ABCG2 | na | 30950 | -0.219 | -0.0642 | No |
| 42 | ABCG8 | na | 31597 | -0.244 | -0.0420 | No |
| 43 | ABCA13 | na | 32767 | -0.305 | -0.0245 | No |
| 44 | ABCC4 | na | 34335 | -0.484 | 0.0113 | No |
Table: GSEA details [plain text format]

  

Fig 2: KEGG\_ABC\_TRANSPORTERS      
 Blue-Pink O' Gram in the Space of the Analyzed GeneSet

  

Fig 3: KEGG\_ABC\_TRANSPORTERS: Random ES distribution      
 Gene set null distribution of ES for **KEGG\_ABC\_TRANSPORTERS**
